# Interleukin‐6 receptor alpha and CD27 discriminate intratumoral T helper 17 subpopulations with distinct functional properties in a mouse lung cancer model

**DOI:** 10.1002/iid3.533

**Published:** 2021-09-27

**Authors:** Chong Liu, Fei Xiong, Lingying Wang, Kang Chen, Pingshang Wu, Li Hua, Zhuo Zhang

**Affiliations:** ^1^ The Department of Thoracic Surgery Wuhan Third Hospital (Tongren Hospital of Wuhan University) Wuhan Hubei Province China

**Keywords:** CD27, interleukin‐6 receptor alpha chain, lung cancer, T helper 17 cells

## Abstract

**Background:**

T helper 17 (Th17) cells actively participate in the tumor immune response in lung cancer. However, the heterogeneity and plasticity of intratumoral Th17 cells in lung cancer remain elusive. In this study, Th17 subpopulations were characterized in a mouse lung cancer model.

**Methods:**

Urethane was administered to induce lung cancer in interleukin (IL)‐17‐EGFP transgenic mice. Flow cytometry was used to analyze the phenotypes, signaling status, and functions of Th17 subpopulations either in vivo or in vitro. The adoptive transfer assay and real‐time polymerase chain reaction were applied to analyze the plasticity of Th17 subpopulations.

**Results:**

IL‐6Rα^high^CD27^−^ Th17 and IL‐6Rα^low^CD27^+^ Th17 were identified in intratumoral Th17 cells. The two subpopulations expressed equivalent RORγt. However, the former expressed higher T‐bet but lower Foxp3, more IL‐17A and IFN‐γ but less IL‐10 than the latter. Furthermore, IL‐6Rα^high^CD27^−^ Th17 moderately inhibited the proliferation of lung cancer cells while IL‐6Rα^low^CD27^+^ Th17 could not. IL‐6Rα^high^CD27^−^ Th17 exhibited weaker Jun N‐terminal kinases (JNK) signaling but stronger signal transducer and activator of transcription 3 (Stat3) signaling than IL‐6Rα^low^CD27^+^ Th17. The adoptive transfer assay indicated that both subpopulations downregulated RORγt in recipients' spleens but maintained RORγt in recipients' lungs. Meanwhile, IL‐6Rα^high^CD27^−^ Th17 expressed higher T‐bet and IFN‐γ than IL‐6Rα^low^CD27^+^ Th17 in the recipients. IL‐6Rα^low^CD27^+^ Th17 expressed Foxp3 and IL‐10 in recipients' spleens but not lungs.

**Conclusions:**

This study reveals intratumoral Th17 subpopulations with distinct functional properties and signaling patterns, thus offering valuable insight into Th17 heterogeneity and plasticity in lung cancer.

## Graphical abstract

IL‐6RαhighCD27^−^ Th17 and IL‐6RαlowCD27+ Th17 cells differentially express master regulators key to T helper cell polarization, as well as cytokines crucial for tumor immunity.

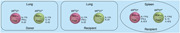

## 1 INTRODUCTION

As one of the most frequent cancers worldwide, lung cancer is a major cause of cancer‐related deaths. The interactions between malignant cells and the associated immune microenvironment are crucial for lung cancer initiation, progression, and metastasis.^1^ Diverse immune cells, including T lymphocytes, macrophages, natural killer cells, dendritic cells, etc., work together to decide whether cancer cells are eradicated, confined, or allowed to grow and migrate to surrounding tissues and distant organs. Both innate and adaptive immunity play vital roles in tumor surveillance and evasion.^2^ T lymphocytes are an important player in mounting a specific immune response against malignant cells. However, T lymphocytes are heterogeneous constituting an array of subsets with distinct functions and regulatory mechanisms. A deep understanding of the roles of T lymphocyte subsets in lung cancer development is of great importance for elucidating the mechanisms of immune evasion of lung cancer and developing efficacious anticancer therapies.

T helper 17 (Th17) cells, a subset of CD4^+^ T lymphocytes that produce interleukin (IL)‐17A, IL‐17F, IL‐22, and other cytokines, are recruited to cancer lesions and inflammatory spots under the guidance of C‐C motif chemokine ligand 20 secreted by epithelium and stromal cells.^3^ The role of Th17 cells in lung cancer immunity has yet to be elucidated, with confusing and contradictory findings in the past decade. For example, IL‐17A was shown to be either protumorigenic or anti‐tumorigenic in different non‐small cell lung cancer (NSCLC) models.^4^, ^5^ Th17 cells might promote tumor cell growth in *K‐ras*‐driven NSCLC while suppressing tumor growth in non‐*K‐ras*‐driven NSCLC.^5^, ^6^ Moreover, cumulative evidence suggests that Th17 cells are a heterogeneous population due to the plasticity of Th17 cells.^7^ The ultimate effect of Th17 cells on lung cancer is perhaps dependent on the dynamics of the proportions and functions of Th17 subpopulations. Therefore, dissection and characterization of Th17 subpopulations in lung cancer lesions would help understand the immune landscape of lung cancer.

For the first time in this study, we unveiled two Th17 subpopulations in a urethane‐induced mouse lung cancer model. Based on the expression of IL‐6 receptor alpha chain (IL‐6Rα) and CD27, intratumoral Th17 cells could be recognized as two subpopulations: IL‐6Rα^high^CD27^−^ Th17 and IL‐6Rα^low^CD27^+^ Th17. The two subpopulations differentially expressed master regulators key to T helper cell polarization, as well as cytokines crucial for tumor immunity. Their functions were, therefore, distinct both in vitro and in vivo. Our study thus deepens the understanding of the heterogeneity of intratumoral Th17 cells in lung cancer.

## 2 MATERIALS AND METHODS

### 2.1 Urethane‐induced lung cancer model

This study was approved by the Wuhan University Animal Care and Use Committee and carried out complying with the Wuhan University Animal Use Guidelines. Adult (8–10 week old) male C57BL/6‐Il17a^tm1Bcgen^ mice, that is, IL‐17A‐EGFP transgenic mice were obtained from Beijing Biocytogen Inc. The model was established based on a published protocol with minor modifications.^8^ Briefly, each mice was intraperitoneally injected with 1 mg of urethane (Sigma‐Aldrich U2500, freshly prepared in 0.9% saline) per gram of body weight on Monday and Thursday every week for 10 weeks. Mice were then euthanized by CO_2_ inhalation and subjected to intracardiac perfusion with 5 ml of ice‐cold phosphate‐buffered saline (PBS). After this, the lung was taken and placed in a 35‐mm petri dish containing 1 ml of ice‐cold (PBS) for further processing.

### 2.2 Preparation of mononuclear cells in lung cancer lesions

The cancer lesions in mouse lungs were carefully separated from surrounding tissues using dissecting scissors and tweezers with curved fine tips. The lesions were then harvested, cut into about small pieces, and incubated in Roswell Park Memorial Institute (RPMI)1640 medium (R8758) containing 20% fetal bovine serum (FBS, 12306C), 2 mg/ml collagenase IV (C4‐BIOC), 2 mM CaCl_2_ (499609), and 100 U/ml DNase I (10104159001) for 30 min at 37°C while shaking at 40 rpm on an orbital shaker (Beyotime Biotech). All reagents were purchased from Sigma‐Aldrich. The digested cancer lesions and associated supernatants were pressed through a 40‐µm pore‐size cell strainer (Beyotime Biotech) to prepare single‐cell suspensions. The single cells were then overlaid onto the Mouse Lymphocyte Separation Media (Liankebio Inc, 70‐MLSM1092) followed by centrifugation at 400 g for 15 min. Mononuclear cells between the interface were harvested and washed with ice‐cold PBS once before flow cytometry analysis.

### 2.3 Flow cytometry analysis

The following antibodies were obtained from Biolegend: Pacific blue‐conjugated anti‐CD3 antibody (17A2), PE/Cy7‐conjugated anti‐CD4 (RM4‐5) antibody, PE anti‐IL‐6Rα (D7715A7), APC/Cy7‐conjugated anti‐CD27 (LG.3A10), APC‐conjugated anti‐IL‐23R (12B2B64), PerCP/Cyanine5.5‐conjugated anti‐IL‐10 (JES5‐16E3), Alexa Fluor® 647‐conjugated anti‐Ki67 (11F6), APC‐conjugated anti‐IFN‐γ (XMG1.2), PE/Cy5‐conjugated anti‐CD44 (IM7), APC‐conjugated anti‐CD62L (MEL‐14). PE anti‐MHC‐II (M5/114.15.2), and APC anti‐MHC‐I (34‐1‐2S) were obtained from eBioscience. Phospho‐JNK (Thr183/Tyr185) Mouse mAb (G9), Phospho‐4E‐BP1 (Thr37/46) Rabbit mAb (236B4), Phospho‐S6 Ribosomal Protein (Ser235/236) XP® Rabbit mAb (D57.2.2E), Phospho‐Stat3 (Tyr705) XP® Rabbit mAb (D3A7) were purchased from Cell Signaling Technology. Anti‐JNK antibody (EPR16797‐211) was bought from Abcam. For Th17 cell detection and sorting, 1 × 10^6^/ml mononuclear cells were incubated with 5 µg/ml corresponding antibodies at 4°C for 15 min. Cells were then washed and with PBS before analysis. For the measurement of intracellular proteins, cells were fixed with 2% paraformaldehyde for 10 min, permeabilized with 90% methanol for 30 min, and stained with 5 µg/ml each corresponding antibody at room temperature for 1 h. If the primary antibody was not conjugated to a fluorochrome, cells were washed with PBS once and stained with 5 µg/ml APC‐conjugated goat anti‐mouse IgG or APC‐conjugated goat antirabbit IgG (both from Abcam) at room temperature for 30 min. For cell death analysis, cells were stained with the APC Annexin V Apoptosis Detection Kit with PI (Biolegend, 640932) following the vendor's protocol. Cells were analyzed on an LSRII flow cytometer or sorted on a FACSAria sorter (both from BD Biosciences).

### 2.4 RNA purification, reverse transcription, and real‐time PCR

Cellular RNAs were purified using the PicoPure® RNA Isolation Kit (Thermo Fisher Scientific, KIT0204). Spleens and cancer nodules were collected from mice, followed by extraction of tissue RNAs using the Trizol reagent (Thermo Fisher Scientific, 15596026) according to the manufacturer's protocol. cDNAs were synthesized using the BeyoRT™ cDNA Synthesis Kit (Bieyotime Biotech, D7178S1). The BlazeTaq™ SYBR® Green qPCR mix (Igenebio, QP071). was used for real‐time PCR on a LightCycler® 480 Instrument II (Roche) following standard procedures. The relative levels of transcripts of interest were normalized to β‐actin and computed by the 2^−ΔΔCt^ equation. Primer sequences are provided in Table S1.

### 2.5 Cell culture and treatment

All reagents were purchased from Sigma‐Aldrich. 1 × 10^5^/ml sorted Th17 subpopulations were cultured in RPMI1640 supplemented with 10% FBS for 6 h with 50 ng/ml phorbol‐12‐myristate‐13‐acetate (PMA, P8139‐1MG) plus 1 µg/ml ionomycin (I0634). Two hours after stimulation, 5 µg/ml brefeldin A (B7651) and 5 µg/ml monensin (M5273) were added into the culture. At the end of stimulation, intracellular staining of cytokines was performed for flow cytometry analysis.

The mouse lung cancer cell line LLC was purchased from and authenticated by Procell. 1 × 10^5^ sorted Th17 subpopulations were activated with PMA plus ionomycin as described above and washed with PBS once, followed by coculture with 1 × 10^4^ LLC cells in a 96‐well plate (Corning). Twenty‐four hours later, cells were incubated with 5 µg/ml pacific blue‐conjugated anti‐CD3 antibody on ice for 15 min, washed with PBS, and then stained with the APC Annexin V Apoptosis Detection Kit with PI or Alexa Fluor® 647‐conjugated anti‐Ki67 antibody as described above. In some experiments, 1 × 10^5^/ml LLC cells were pretreated with 100 ng/ml mouse IFN‐γ (Peprotech, #315–05) for 48 h before coculture.

### 2.6 Adoptive transfer

Sorted Th17 subpopulations were labeled with CellTrace Violet (Thermo Fisher Scientific, C34571) following the vendor's instructions. The Th17 subpopulations were separately infused into lung cancer‐bearing IL‐17A‐EGFP transgenic mice through retro‐orbital injection (2 × 10^6^ cells of each subpopulation in 200 µl of 0.9% saline for each mouse). Three days after transfer, the recipients were euthanized and spleens and lungs were collected. Mononuclear cells in recipients' lungs were isolated as described above. The spleens were ground in a 40‐µm pore size strainer to prepare single splenocyte suspensions. The Violet^+^ exogenous Th17 cells in the mononuclear cells and splenocytes were then discriminated and sorted by flow cytometry for further analysis.

### 2.7 Statistics

All experiments were independently performed two or three times. The data were indicated as mean ± standard deviation. The Student's *t*‐test or one‐way analysis of variance (ANOVA) with Tukey post hoc test was used for statistical analysis. A *p* value less than .05 was regarded as significant.

## 3 RESULTS

### 3.1 IL‐6Rα and CD27 are differentially expressed on intratumoral Th17 cells

**Figure 1:**
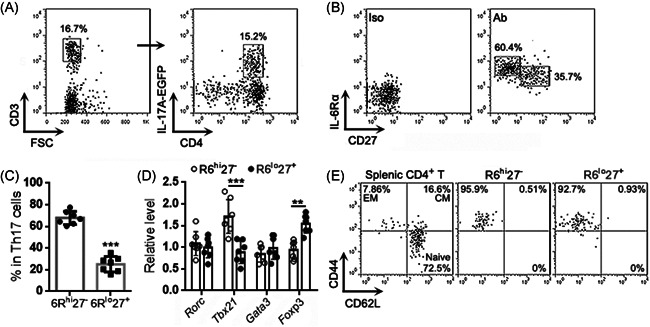
Presence of intratumoral Th17 subpopulations in lung cancer. (A) Gating IL‐17A‐EGFP^+^ Th17 cells among mononuclear cells isolated from lung cancer lesions. (B) Expression of IL‐6Rα and CD27 on Th17 cells. Iso: isotype control. Ab: specific antibodies. (C) Frequencies of subpopulations in total Th17 cells. (D) Transcript levels of indicated master regulators in Th17 subpopulations. (E) Expression of CD44 and CD62L on Th17 subpopulations. Splenic CD4^+^ T, splenic total CD4^+^ T cells; CM, central memory T cells; EM, effector memory T cells; Naïve, naïve T cells. *N *= 7 samples per group. Student's *t*‐test. **p<.01. ***p<.001

To characterize Th17 cells in lung cancer, we established a urethane‐induced lung cancer model in IL‐17A‐EGFP transgenic mice (Figure S1) and isolated cells from lung cancer lesions. Using flow cytometry, singlets were first gated among all events and intact live cells were gated within the singlets (Figure S2). CD3^+^ T cells were gated among live cells, and IL‐17A‐EGFP^+^ Th17 cells were then recognized among T cells (Figure 1A). As indicated in Figure 1B, IL‐6Rα and CD27 were differentially expressed on Th17 cells, showing an IL‐6Rα^high^CD27^‐^ subpopulation (Hereinafter 6R^hi^27^−^) and an IL‐6Rα^low^CD27^+^ subpopulation (Hereinafter 6R^lo^27^+^). The 6R^hi^27^−^ subpopulation was almost twice as many as the 6R^lo^27^+^ subpopulation (Figure 1C). Examination of the mRNA levels of the genes encoding T helper cell master regulators, such as *Tbx21* (encoding T‐bet), *Rorc* (encoding RORγt), *Foxp3* (encoding Foxp3), and *Gata3* (encoding Gata3), revealed equivalent *Rorc* mRNA levels in the two subpopulations (Figure 1D). However, the 6R^hi^27^−^ subpopulation expressed higher *Tbx21* mRNA and lower *Foxp3* mRNA than the 6R^lo^27^+^ subpopulation (Figure 1D), suggesting that the former likely involved Th17/Th1 cells whereas the latter included Treg‐like cells. We also checked the expression of two effector/memory T cell markers, CD44 and CD62L. Both 6R^hi^27^−^ cells and 6R^lo^27^+^ cells were predominantly CD44^+^CD62L^−^, suggesting that they were phenotypically effector memory T cells (Figure 1E).

### 3.2 The two Th17 subpopulations exhibited different functional properties

**Figure 2:**
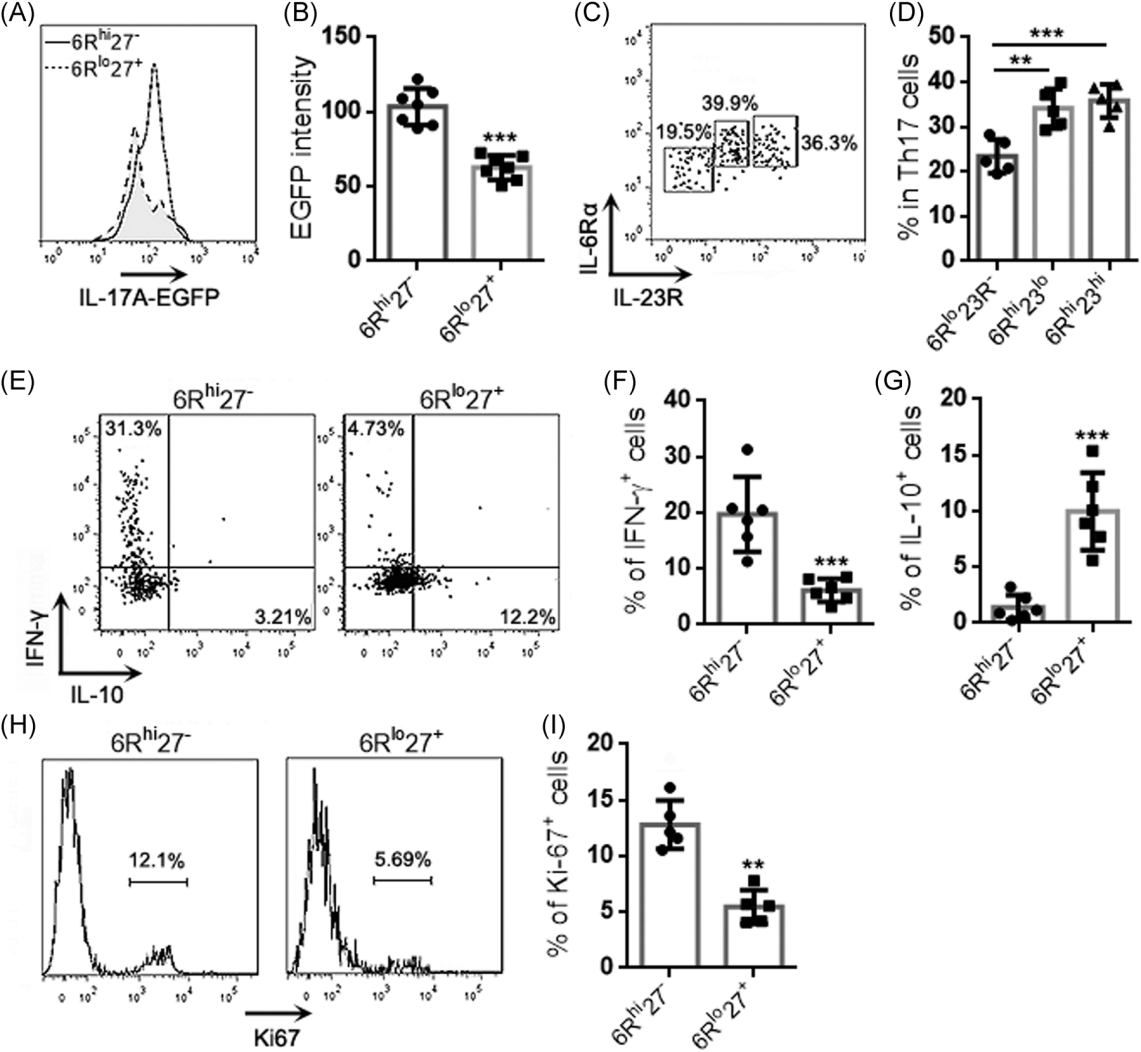
Cytokine expression and proliferation of Th17 subpopulations. (A) and (B) Intensities of IL‐17A‐EGFP in Th17 subpopulations. Representative histograms are shown in (A). Statistics are shown in (B). (C) and (D) IL‐6Rα and IL‐23R expression on total Th17 cells. Representative dot plots are shown in (C). Statistics are shown in (D). (E)–(G) Expression of IFN‐γ and IL‐10 in Th17 subpopulations. Representative dot plots are shown in (E). Statistics are shown in (F) and (G). (H) and (I) Ki‐67 staining in Th17 subpopulations. Representative histograms are shown in (H). Statistics are shown in (I). *N *= 6 or 7 samples per group. Student's *t*‐test. **p<.01. ***p<.001

We also evaluated the intensities of IL‐17A‐EGFP in the two Th17 subpopulations. As shown in Figure 2A,B, the 6R^hi^27^−^ subpopulation possessed a higher IL‐17A‐EGFP intensity than the 6R^lo^27^+^ subpopulation, suggesting more IL‐17A expression in the former. Interestingly, the expression patterns of IL‐23R and IL‐6Rα suggested that the IL‐6Rα^hi^ Th17 expressed higher IL‐23R than the IL‐6Rα^lo^ Th17 and could be further divided into a 6R^hi^27^‐^IL‐23R^lo^ subpopulation and a 6R^hi^27^‐^IL‐23R^hi^ subpopulation (Figure 2C,D). We then assessed the production of IFN‐γ and IL‐10 in the 6R^hi^27^−^ and 6R^lo^27^+^ subpopulations after activation with PMA and ionomycin. As demonstrated in Figure 2E–G, THE 6R^hi^27^−^ subpopulation produced abundant IFN‐γ but very low IL‐10, whereas the 6R^lo^27^+^ subpopulation expressed IL‐10 but fewer IFN‐γ. The Ki‐67 staining suggested a higher proliferative rate of the 6R^hi^27^−^ subpopulation relative to the 6R^lo^27^+^ subpopulation (Figure 2H,I).

**Figure 3:**
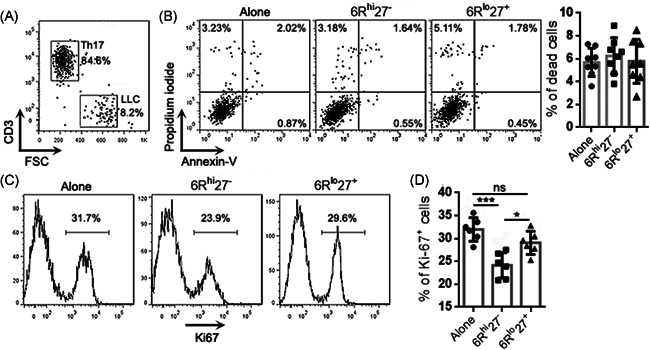
Inhibitory effects of Th17 subpopulations on lung cancer cells. (A) Recognition of LLC cells after coculture with the 6R^hi^27^−^ Th17 subpopulation. A similar analysis was done on LLC cells cocultured with the 6R^lo^27^+^ Th17 subpopulation. (B) Apoptosis and necrosis of LLC cells. Alone: LLC cells cultured alone. 6R^hi^27^−^: coculture with 6R^hi^27^−^ Th17. 6R^lo^27^+^: coculture with 6R^lo^27^+^ Th17. Left panel: representative dot plots. Right panel: statistics of dead (apoptotic plus necrotic) LLC cells. (C) and (D) Ki‐67 staining in LLC cells. Representative histograms are shown in (C). Statistics of the percentages of Ki‐67^+^ LLC cells are shown in (D). *N *= 6 or 8 samples per group. One‐way ANOVA. *p<.05. ***p<.001. ns, not significant

To further analyze the functions of the two subpopulations, 6R^hi^27^−^ cells and 6R^lo^27^+^ cells were activated with PMA plus ionomycin and then cocultured with the mouse lung cancer cell line LLC (effector‐target ratio = 10:1) for 24 h (Figure 3A). Neither 6R^hi^27^−^ cells nor 6R^lo^27^+^ cells significantly induced LLC cell death, because the frequencies of dead LLC cells after coculture were comparable with the frequency of LLC cells undergoing spontaneous death (Figure 3B). However, 6R^hi^27^−^ cells were more inhibitory than 6R^lo^27^+^ cells to LLC cell proliferation, as evidenced by lower Ki‐67 expression in LLC cells that were cocultured with 6R^hi^27^−^ cells (Figure 3C,D). 6R^lo^27^+^ cells did not inhibit LLC cell proliferation (Figure 3C,D). To test whether the effect of 6R^lo^27^+^ cells was MHC‐dependent, LLC cells were stimulated with IFN‐γ for 48 h. The stimulation markedly upregulated MHC‐I expression but did not induce MHC‐II expression on LLC cells (Figure 3A). Stimulated LLC cells were then cocultured with PMA‐activated 6R^hi^27^−^ cells or 6R^lo^27^+^ cells as described above. 6R^hi^27^−^ cells caused the equivalent reduction of Ki67 expression in unstimulated LLC and stimulated LLC, suggesting that the inhibitory effect of 6R^hi^27^−^ cells was MHC‐independent (Figure 3B).

### 3.3 The two Th17 subpopulations exhibited differential signaling status

**Figure 4:**
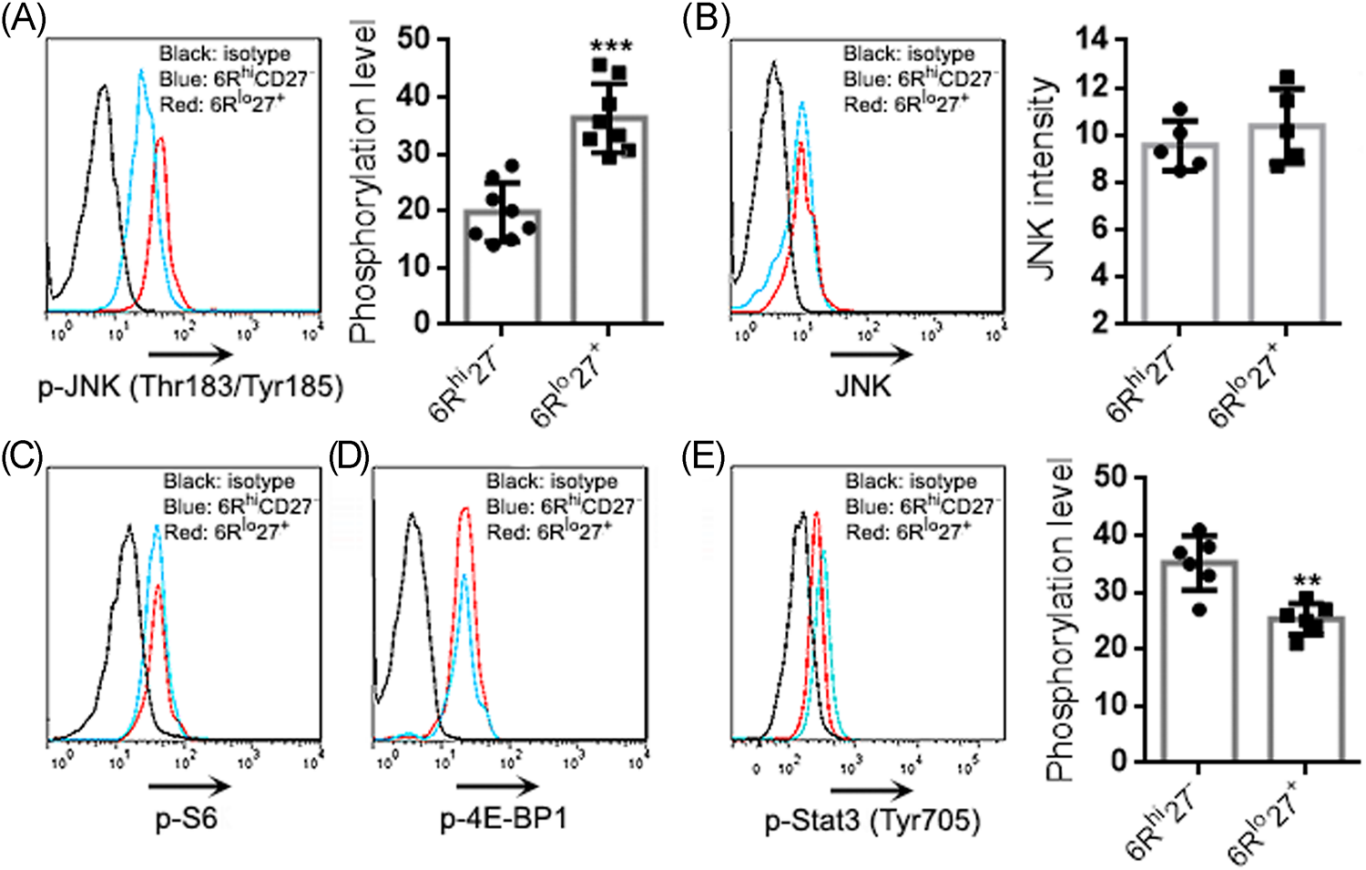
Signaling status of Th17 subpopulations. (A) JNK phosphorylation in Th17 subpopulations. Left panel: representative histograms of phosphorylated JNK. Right panel: statistics of the intensity of phosphorylated JNK. (B) JNK expression in Th17 subpopulations. Left panel: representative histograms of JNK. Right panel: statistics of the intensity of JNK. (C) and (D) Phosphorylation of S6 and 4E‐BP1 in Th17 subpopulations. The data represent two independent experiments. (E) Stat3 phosphorylation in Th17 subpopulations. Left panel: representative histograms of phosphorylated Stat3. Right panel: statistics of the intensity of phosphorylated Stat3. *N *= 5–8 samples per group. Student's *t*‐test. **p<.01. ***p<.001

It has been reported that CD27 mediates the Jun N‐terminal kinases (JNK) signaling and CD27^+^ Th7 cells exhibited weak mechanistic target of rapamycin complex 1 (mTORC1) signaling.^9^, ^10^ To check if this was also the case in our model, we measured the phosphorylation of JNK, the ribosomal protein S6 (a mTORC1 substrate), and 4E‐BP1 (another mTORC1 substrate). As shown in Figure 4A, 6R^hi^27^−^ cells had fewer phosphorylated JNK than 6R^lo^27^+^ cells but they had equivalent total JNK expression (Figure 4B). However, no differences in either phosphorylated S6 or phosphorylated 4E‐BP1 were found between the two subpopulations (Figure 4C,D). Interestingly, 6R^hi^27^−^ cells had more phosphorylated Stat3 than 6R^lo^27^+^ cells (Figure 4E), suggesting stronger Stat3 signaling in 6R^hi^27^−^ cells.

### 3.4 The two Th17 subpopulations exhibited differential expression of master regulators after adoptive transfer

**Figure 5:**
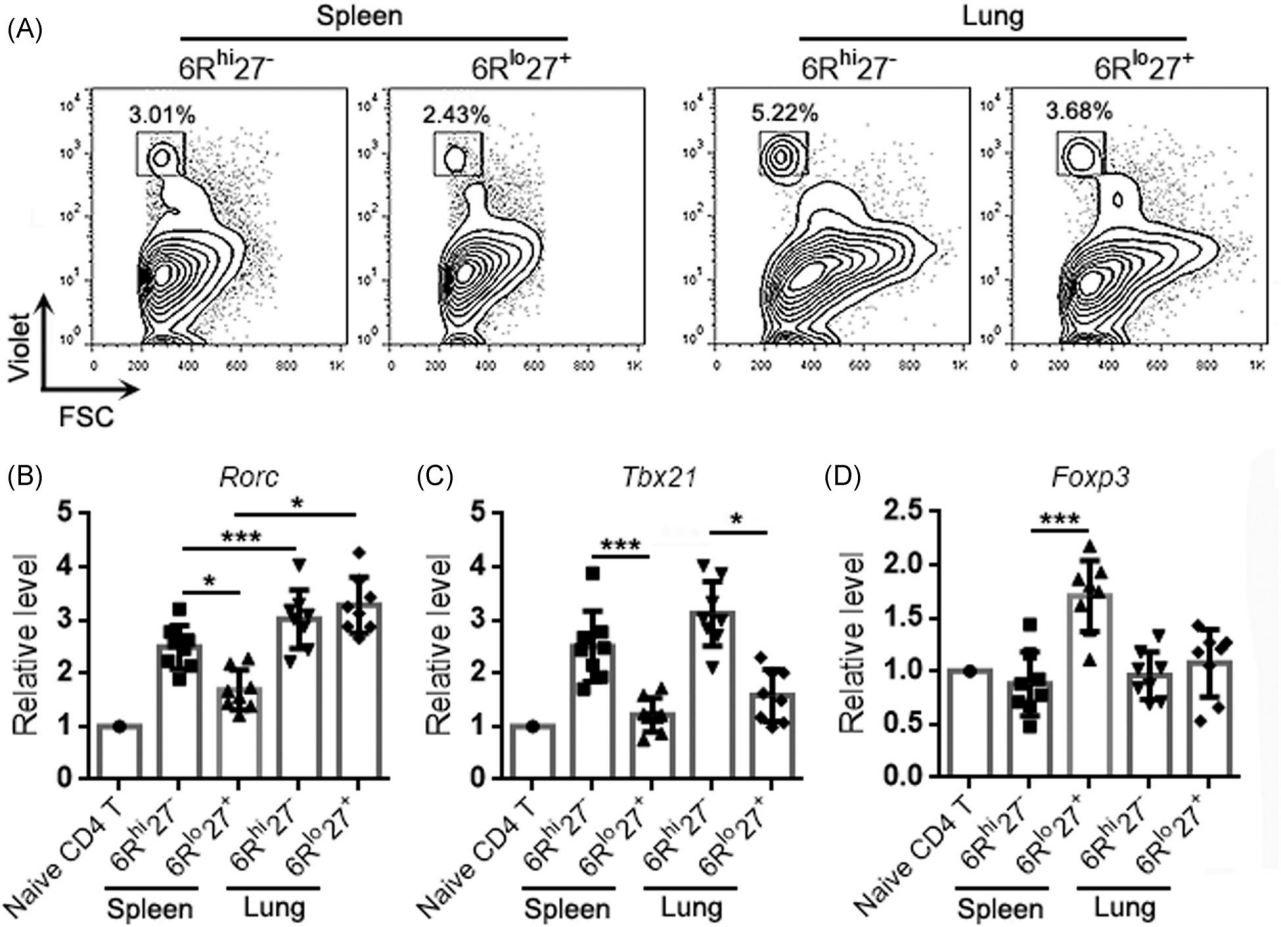
Expression of master regulators in adoptively transferred Th17 subpopulations. (A) Recognition of exogenous Violet^+^ Th17 subpopulations in recipients’ spleens and lungs. (B)–(D) Transcript levels of indicated master regulators in exogenous Th17 subpopulations. Naïve CD4 T: CD4^+^ T cells sorted from a naïve mouse as a control. *N *= 8 samples per group. One‐way ANOVA. *p<.05. ***p<.001. ANOVA, analysis of variance

To characterize the differentiation properties of the two Th17 subpopulations, 6R^hi^27^−^ Th17 cells and 6R^lo^27^+^ Th17 cells were sorted, labeled with CellTrace Violet, and then adoptively infused into mice bearing lung cancer. Three days later, Violet^+^ exogenous Th17 cells were retrieved from recipients' spleens and lungs for analysis (Figure 5A). Exogenous 6R^hi^27^−^ Th17 cells expressed higher *Rorc* mRNA than 6R^lo^27^+^ Th17 cells in recipients' spleens but the two subpopulations expressed equivalent *Rorc* mRNA in recipients' lungs (Figure 5B). Interestingly, the two subpopulations in the lung expressed higher *Rorc* mRNA than their respective counterparts in recipients' spleens (Figure 5B). Further evaluation revealed that 6R^hi^27^−^ Th17 cells expressed higher *Tbx21* mRNA than 6R^lo^27^+^ Th17 cells in recipients' spleens and lungs, and there was no significant difference in T‐bet expression levels between Th17 cells in recipients' spleens and their counterparts in recipients' lungs (Figure 5C). 6R^lo^27^+^ Th17 cells expressed *Foxp3* mRNA in recipients' spleens but not in recipients' lungs (Figure 5D).

### 3.5 The two Th17 subpopulations exhibited differential cytokine expression after transfer

**Figure 6:**
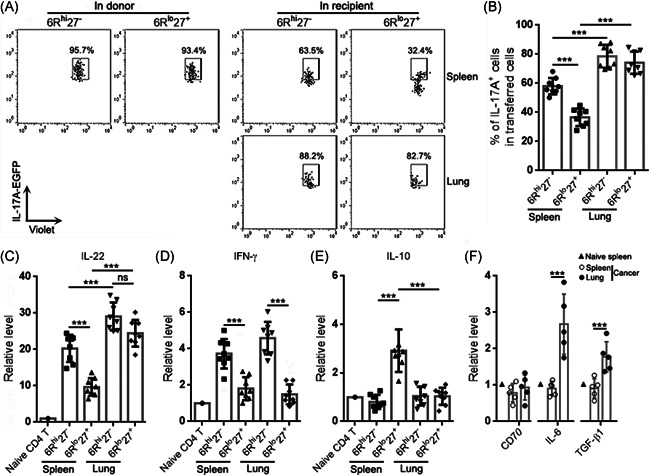
Cytokine expression in adoptively transferred Th17 subpopulations. (A) Representative dot plots showing IL‐17A‐EGFP^+^ cells in exogenous Th17 subpopulations. In donor: Th17 subpopulations in donors' lungs. In recipients: Th17 subpopulations in recipients. (B) Statistics of the percentages of IL‐17A‐EGFP^+^ cells in exogenous Th17 subpopulations in recipients' spleens and lungs. (C)–(E) Transcript levels of indicated cytokines in exogenous Th17 subpopulations in recipients' spleens and lungs. Naïve CD4 T: CD4^+^ T cells sorted from a naïve mouse as a control. (F) Transcript levels of indicated molecules in spleens and cancer nodules. Naïve spleen: spleen of a naïve mice. Cancer, cancer‐bearing mice. Lung, lung cancer nodules. *N *= 5 or 8 samples per group. One‐way ANOVA. ***p < .001. ANOVA, analysis of variance; ns, not significant.

**Figure 7:**
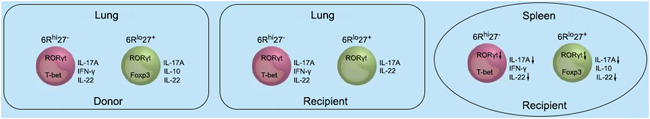
Summary of the features of Th17 subpopulations in donors and recipients. Arrow: downregulation

We also determined the proportions of IL‐17A‐EGFP^+^ cells in the transferred Th17 subpopulations. In donors, more than 90% of Th17 cells were IL‐17A‐EGFP^+^. In recipients' spleens, IL‐17A‐EGFP^+^ cells accounted for 60% of exogenous 6R^hi^27^−^ Th17 cells and 40% of exogenous 6R^lo^27^+^ Th17 cells, respectively (Figure 6A,B). However, in recipients' lungs, IL‐17A‐EGFP^+^ cells accounted for 80% of either exogenous 6R^hi^27^−^ or 6R^lo^27^+^ Th17 cells (Figure 6A,B). The transcript levels of IL‐22 showed a similar change to IL‐17A. Exogenous 6R^hi^27^−^ Th17 cells expressed more IL‐22 than exogenous 6R^lo^27^+^ Th17 cells in recipients' spleens, while both subpopulations in recipients' spleens expressed less IL‐22 than their counterparts in recipients' lungs (Figure 6C). Consistent with the T‐bet expression pattern presented in Figure 5C, exogenous 6R^hi^27^−^ Th17 cells expressed higher IFN‐γ than exogenous 6R^lo^27^+^ Th17 cells in both recipients' spleens and lungs. There was no significant difference in IFN‐γ expression between exogenous Th17 cells in recipients' spleens and their counterparts in recipients' lungs (Figure 6D). Consistent with the Foxp3 expression pattern presented in Figure 5D, exogenous 6R^lo^27^+^ Th17 cells expressed IL‐10 in recipients' spleens but not in recipients' lungs (Figure 6E). Figure 7 summarizes the functional features of the two subpopulations in donors and recipients.

To check whether the cancer microenvironment harbored factors that could influence Th17 subpopulations, we evaluated the mRNA levels of CD70 (the CD27 ligand), IL‐6, and TGF‐β1 in the lysates of cancer nodules and spleens. As illustrated in Figure 6F, compared with the naïve spleen and spleens of cancer‐bearing mice, CD70 was unchanged in cancer nodules. However, IL‐6 and TGF‐β1 were upregulated in cancer nodules.

Regardless of the above changes, the lung cancer progression and mouse survival were not remarkably altered (data not shown), suggesting that the adoptive infusion was incapable of altering anti‐tumor immunity. A more efficient approach is needed to determine the roles of the two subpopulations in lung cancer development.

## 4 DISCUSSION

The present study showed two intratumoral Th17 subpopulations in mice with lung cancer. Although the heterogeneity of Th17 cells has been recognized in the past decade, this is the first report describing intratumoral Th17 subpopulations based on the expression of IL‐6Rα and CD27. IL‐6Rα is part of the receptor for IL‐6. It binds to IL‐6 with low affinity and induces dimerization of gp130 to initiates intracellular signaling.^11^ IL‐6R is expressed in Th17 cells and is necessary for retaining Th17‐related transcriptional and functional profiles.^12^, ^13^ According to our data, IL‐6Rα was differentially expressed on intratumoral Th17 cells, with one subpopulation expressing higher IL‐6Rα and another subpopulation expressing relatively lower IL‐6Rα. However, it is still unclear what caused this discrepancy in IL‐6Rα expression. It is possible that some Th17 cells downregulated IL‐6Rα after binding to IL‐6, because the activation of the signaling complex leads to IL‐6/IL‐6R/gp130‐complex.^14^ Nonetheless, IL‐6 also induces IL‐6R upregulation and enhances recycle of IL‐6Rα and gp130 back to the cell surface after internalization and activation of signaling.^15^, ^16^ Therefore, the exact cause of the differential IL‐6Rα expression needs further investigations. Consistent with the well‐known IL‐6/IL‐6R‐mediated cell signaling, the IL‐6Rα^hi^ Th17 subpopulation exhibited higher Stat3 phosphorylation, suggesting the higher IL‐6Rα expression is related to stronger activation of the Stat3 signaling.

The differential CD27 expression may also directly correlates to the distinct functions of Th17 subpopulations. Upon binding to its ligand CD70, CD27 promotes JNK phosphorylation to antagonize RORγt‐induced IL‐17A and IL‐17F via transcriptional suppression or epigenetically silencing the *Il17a* locus.^17^ Indeed, we found elevated JNK phosphorylation in 6R^lo^27^+^ Th17 cells and thus concluded that CD27 suppressed the pro‐inflammatory function of 6R^lo^27^+^ Th17 cells in lung cancer lesions. However, similar to the question above, the factors responsible for the differential CD27 expression remain unidentified. CD27 is upregulated on naïve T cells upon activation but downregulated after differentiation into effector T cells, whereas memory T cells re‐express CD27. Therefore, 6R^lo^27^+^ Th17 cells might be memory Th17 cells. Indeed, a recent study already indicates that CD27⁺ Th17 cells have memory‐like features.^10^

Interestingly, the expression patterns of T‐bet and Foxp3, as well as IFN‐γ and IL‐10 suggest that the 6R^hi^27^−^ subpopulation harbored Th17/Th1 cells while the 6R^lo^27^+^ subpopulation contained Treg‐like cells. This finding indicates the complicated Th17 plasticity in lung cancer. Although both Th17 subpopulations could not kill lung cancer cell line LLC, the 6R^hi^27^−^ subpopulation exhibited a moderate inhibitory effect on LLC proliferation, probably due to their production of IFN‐γ. In the future, it will be interesting to evaluate the potential immunosuppressive effect of the 6R^lo^27^+^ subpopulation.

Under the stimulation of PMA, about 20% of 6R^hi^27^−^ cells expressed IFN‐γ while only 10% of 6R^lo^27^+^ cells expressed IL‐10, suggesting that these two subpopulations might also be heterogeneous and could be further divided into more subpopulations. This assumption is supported by the data showing that IL‐6Rα^hi^ Th17 cells consisted of a 6R^hi^27^‐^IL‐23R^lo^ subpopulation and a 6R^hi^27^‐^IL‐23R^hi^ subpopulation (Figure 2C). IL‐6Rα^lo^ Th17 cells could also be dissected into distinct subpopulations based on surface or genetic markers in future investigations. Furthermore, the adoptive transfer assay showed a more intriguing Th17 plasticity. After summarizing the data in Figures 5 and 6, it is possible that (1) lung cancer induced the expression of RORγt mRNA in both subpopulations (Figure 5B). This might be due to the effects of IL‐6 and TGF‐β1 in the cancer sites. The upregulation of RORγt was associated with an increase in IL‐22 (Figure 6C). Hence, the cancer sites could reinforce Th17 differentiation; (2) Foxp3 was downregulated in the 6R^lo^27^+^ subpopulation in the lung (Figure 5D). So, this subpopulation might be less immunosuppressive in the cancer sites. And this is supported by the change in IL‐10 (Figure 6E); (3) T‐bet and IFN‐γ were unchanged in the spleen and in the lung, suggesting no effect of the cancer microenvironment on the “Th1” response of Th17 cells.

In the future, using conditional knock‐in and knockout mouse strains or lineage tracing models, the real identities and functions of the two subpopulations will be disclosed and more Th17 subpopulations would be revealed. Our research thus opens a new gate for classifying and elucidating Th17 heterogeneity and plasticity in lung cancer.

## CONFLICT OF INTERESTS

The authors declare that there are no conflict of interests.

## AUTHOR CONTRIBUTIONS

Zhuo Zhang designed and supervised the research. Chong Liu, Fei Xiong, and Lingying Wang did most experiments. Kang Cheng carried out the in vitro culture experiment. Pingshang Wu and Li Hua did real‐time PCR.

## Supporting information

Supporting information.

## Data Availability

The data sets generated during and/or analyzed during the current study are available from the corresponding author on reasonable request.
