# Supplementary material for: Interleukin‐6 receptor alpha and CD27 discriminate intratumoral T helper 17 subpopulations with distinct functional properties in a mouse lung cancer model
Source: Immun Inflamm Dis. 2021 Sep 27;9(4):1749–58. doi: 10.1002/iid3.533 (PMC8589402; doi:10.1002/iid3.533)
Supplement: Supplementary file 1 — Supporting information. [file IID3-9-1749-s001.docx]

| **Supplementary Table 1. Real-time PCR primers** | | |
| --- | --- | --- |
| Target | Forward (5’ to 3’) | Reverse (5’ to 3’) |
| RORγt | gtggagtttgccaagcggcttt | cctgcacattctgactaggacg |
| T-bet | ccacctgttgtggtccaagttc | ccacaaacatcctgtaatggcttg |
| Gata3 | cctctggaggaggaacgctaat | gtttcgggtctggatgccttct |
| Foxp3 | tacttcaagttccacaacatggg | cacaaagcacttgtgcagactcag |
| INF-γ | gcgtcattgaatcacacctga | tgtgggttgttgacctcaaact |
| IL-22 | tttcctcgtcggcttgctctgt | cgtgttcttggatgaagcgtagg |
| CD70 | gcggactactcagtaagcagca | tgtgaaggaccttcccaaggct |
| IL-6 | taccacttcacaagtcggaggc | ctgcaagtgcatcatcgttgttc |
| TGF-β1 | tgatacgcctgagtggctgtct | cacaagagcagtgagcgctgaa |
| β-Actin | cattgctgacaggatgcagaagg | tgctggaaggtggacagtgagg |


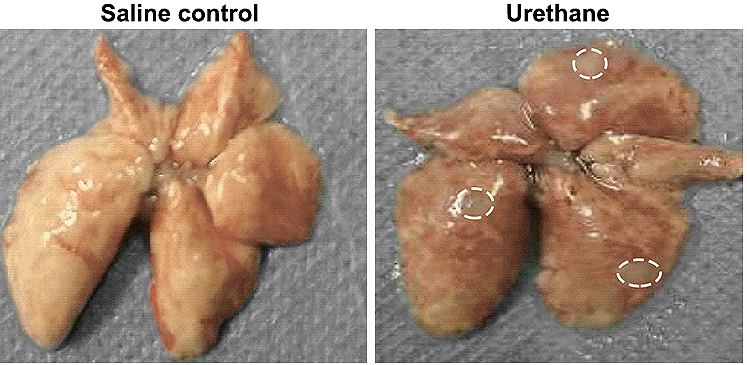


**Supplementary Figure 1. Morphology of the lung tissue in a saline-administered mouse (Left) and urethane-administered mouse (Right).** The dashed circles indicate lung cancer lesions.


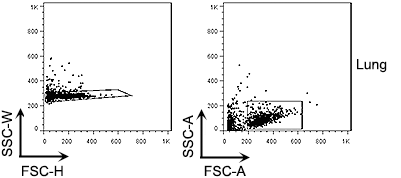


**Supplementary Figure 2. Gating singlets and live cells in mononuclear cells that were isolated from lung cancer lesions.**

**
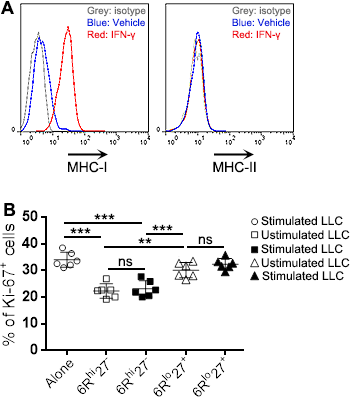
**

**Supplementary Figure 3. The inhibitory effect of 6R^hi^27^-^ cells is MHC-independent. (A)** The expression of MHC-I and MHC-II in LLC cells. Isotype: isotype antibody control. Vehicle: PBS treatment. IFN-γ: IFN-γ stimulation. **(B)** Ki67 expression in LLC cells after co-culture with Th17 subsets. Unstimulated LLC: PBS-treated LLC. Stimulated LLC: IFN-γ stimulated LLC. N=6 samples per group. One-way ANOVA.
